# An Unorthodox Skin Traction Technique for the Initial Management of Distal Femur Fractures in a High-volume Trauma Centre: A Technical Review

**DOI:** 10.7759/cureus.7106

**Published:** 2020-02-26

**Authors:** Andrea Mc Carthy, Eoghan Meagher, Mark Dolan

**Affiliations:** 1 Orthopaedics, Cork University Hospital, Cork, IRL

**Keywords:** femur fracture, traction, pre-operative management, pain, deformity

## Abstract

Distal femur fractures account for 3% of femur fractures and require definitive fixation to allow for weight-bearing and return of functional capability. However, if these fractures must wait a period of time to be taken to theatre, skin traction is routinely applied in the pre-operative period to maximise pain management, prevent deformity and protect neurovascular status. Pre-made traction kits are usually widely available in emergency departments worldwide, allowing for the rapid application and stabilisation of the limb once analgesia in the form of a femoral block has been delivered.

Unfortunately, as in many aspects of healthcare, demand can sometimes outweigh supply. In high-volume-trauma centres or mass-casualty incidents, the pre-made kits designed for skin traction such as Sterotrac (Steroplast Healthcare, Manchester, UK) or Tensoplast (BSN medical GmbH, Hamburg, Germany) kits can be rapidly depleted, leaving emergency and orthopaedic physicians with no means of providing the traction required. Hence, we propose and describe a modified technique that provides a simple and inexpensive way to achieve and maintain skin traction using readily available hospital supplies, which can provide adequate support in a safe manner until definitive surgical fixation. This method not only provides sufficient traction but protects the bony pressure areas around the foot and ankle, thereby reducing the risk of iatrogenic pressure sores.

## Introduction

Distal femur fractures account for approximately 3% of all femoral fractures with two demographics representing epidemiologically defined peaks: young men in their thirties and older females [1-2]. The younger cohort often sustains the injury as part of high-energy, polytrauma-inducing accidents while the older cohort typically sustains spiroid fragility fractures through osteoporotic bone usually following a fall [1-2]. Fixation is necessary for both cohorts and, pending surgery, balanced traction is recommended to optimise pain management and prevent excessive exertion of force through the skin and overlying soft tissue layers [3-4]. However, skin traction is not without risk, and the most common complications reported include pressure sores and an increased risk of deep vein thrombosis (DVT) [3-4].

Although distal femur fractures are rare, level-one trauma centres or mass casualty situations causing high-volume influxes of patients may experience a depletion of resources, and access to the supply of ready-made skin traction kits or Thomas Splints may be diminished [1-2]. The technique reviewed in this paper provides a simple way to create a skin traction system using readily available resources, and it works as effectively as pre-made kits.

## Technical report

The patient is initially assessed under the principles of Advanced Trauma Life Support (ATLS). The femur fracture will be identified on the radiograph and the specialist orthopaedic team will be informed. Ideally, after the completion of a femoral nerve block for acute analgesia, swift application of a traction device or kit is warranted for sub-acute pain relief and to prevent the aforementioned excessive internal exertional forces applied by displaced fracture segments that can lead to skin necrosis [4-5]. Should a scenario arise wherein ready-made skin traction kits or Thomas Splint kits are unavailable, a simple method using common hospital resources can be employed to produce an effective temporary skin traction kit.

**Figure 1:**
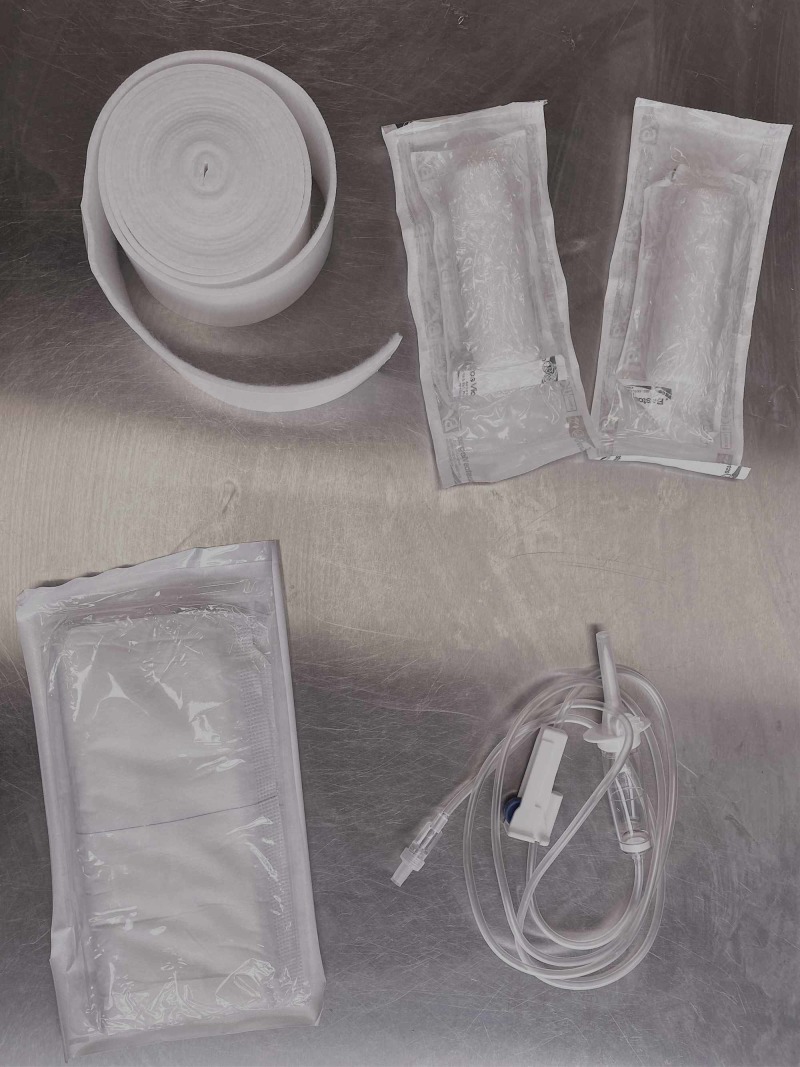
Items required for the self-made traction kit, including orthopaedic felt, crepe bandages, surgical pads and IV tubing

For preparing this kit, we need one roll of adhesive padding, three crepe bandages, shears or scissors, medical tape (zinc oxide), two surgical pads and an IV giving set with bags of IV fluid (the amount will depend on the degree of traction needed and the patient’s body habitus) (Figure 1). As is the case with applying skin traction traditionally, the help of an assistant is required.

**Figure 2:**
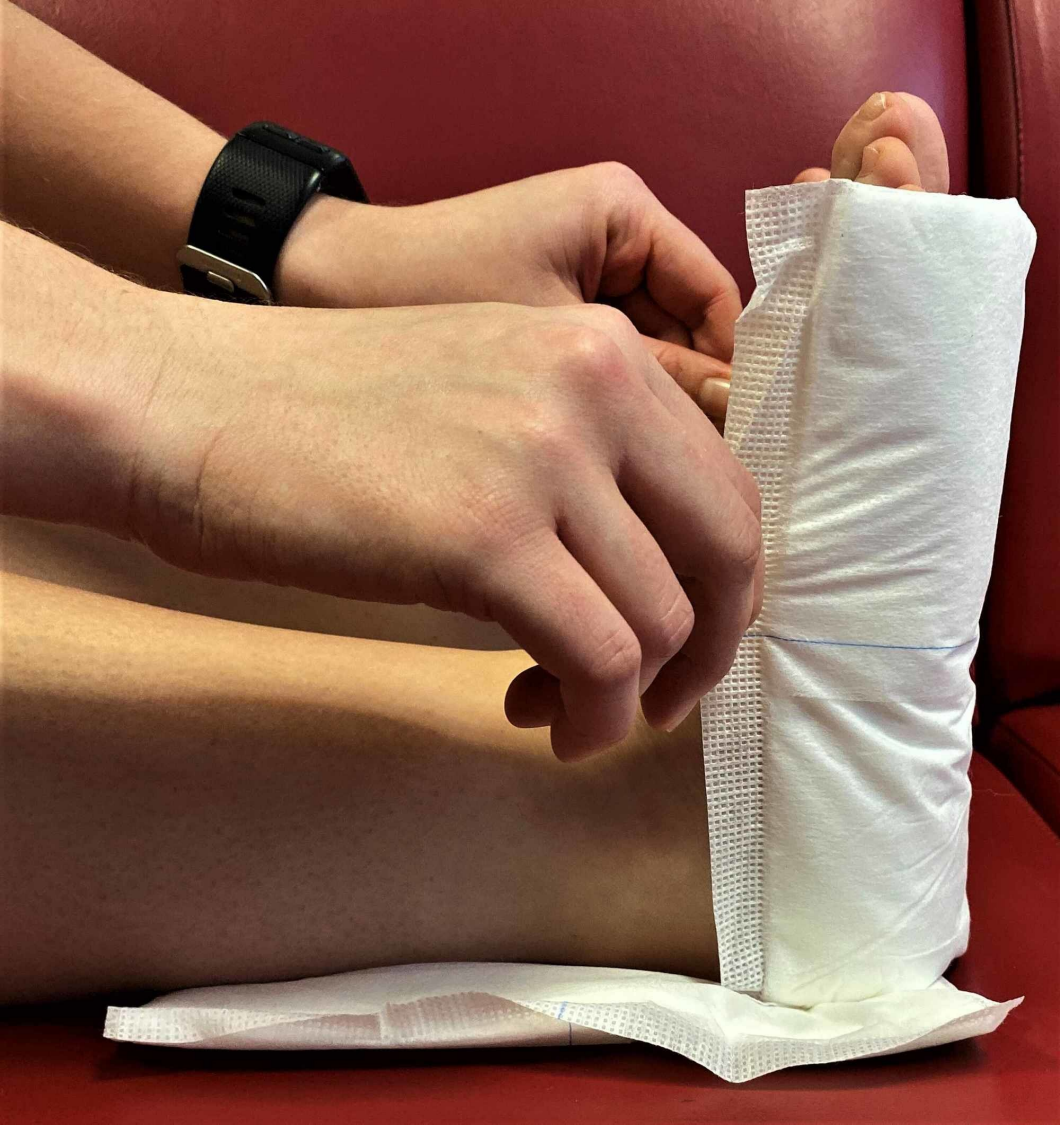
Surgical pads are placed around the Achilles tendon and malleoli and around the heel and base of the foot

The two surgical pads are placed around the foot, one under the foot from the heel to toe and one posteriorly covering the heel and distal Achilles tendon. This will provide padding and absorb sweat preventing moisture build-up that can further contribute to sore development (Figure 2).

**Figure 3:**
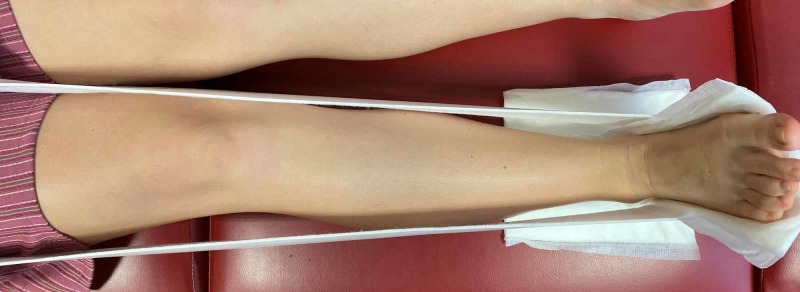
Orthopaedic felt is used to create the traction band

**Figure 4:**
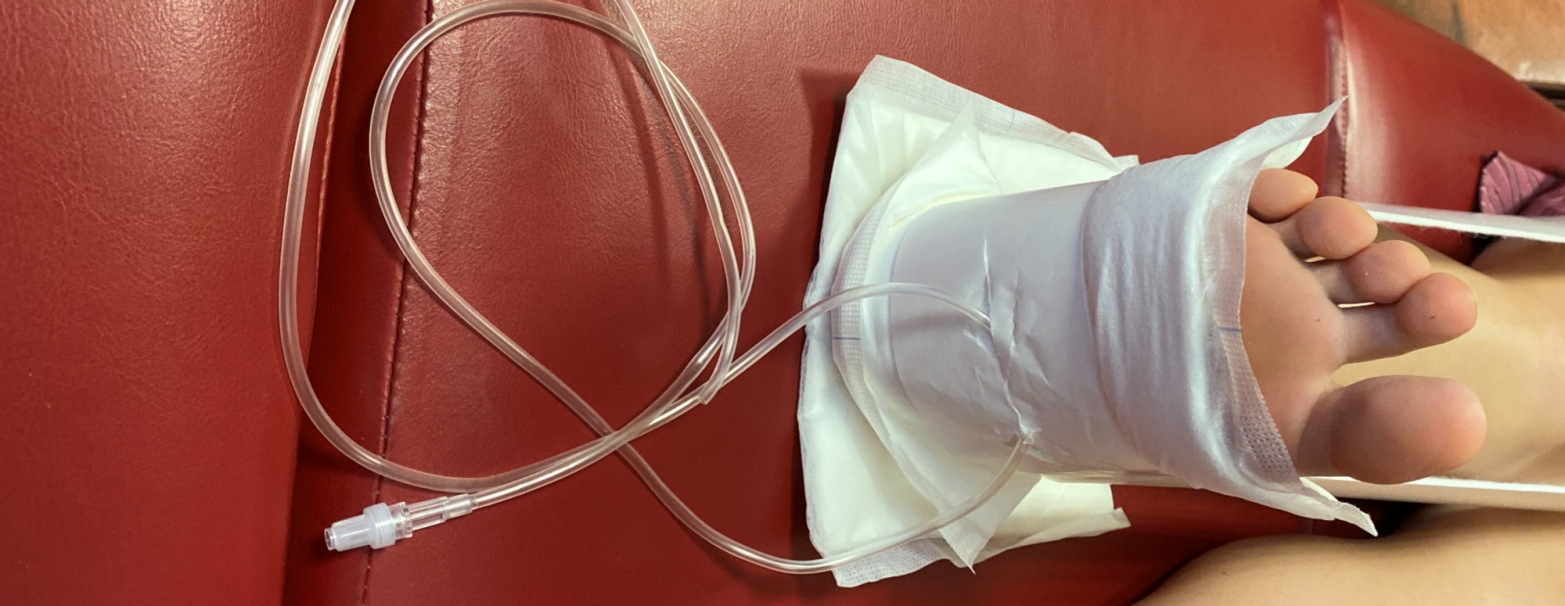
Holes are made at the bottom of the felt with the tubing threaded through

The padding strip is then measured from under the foot and rolled up the leg on either side until it is approximately 10 cm above the knee joint (Figure 3). This length is cut using the shears/scissors and two holes are made in the bottom of the padding strip for the weight (bags of IV fluid) to be hung from (Figure 4). The padding is then secured in place by the assistant.

 

**Figure 5:**
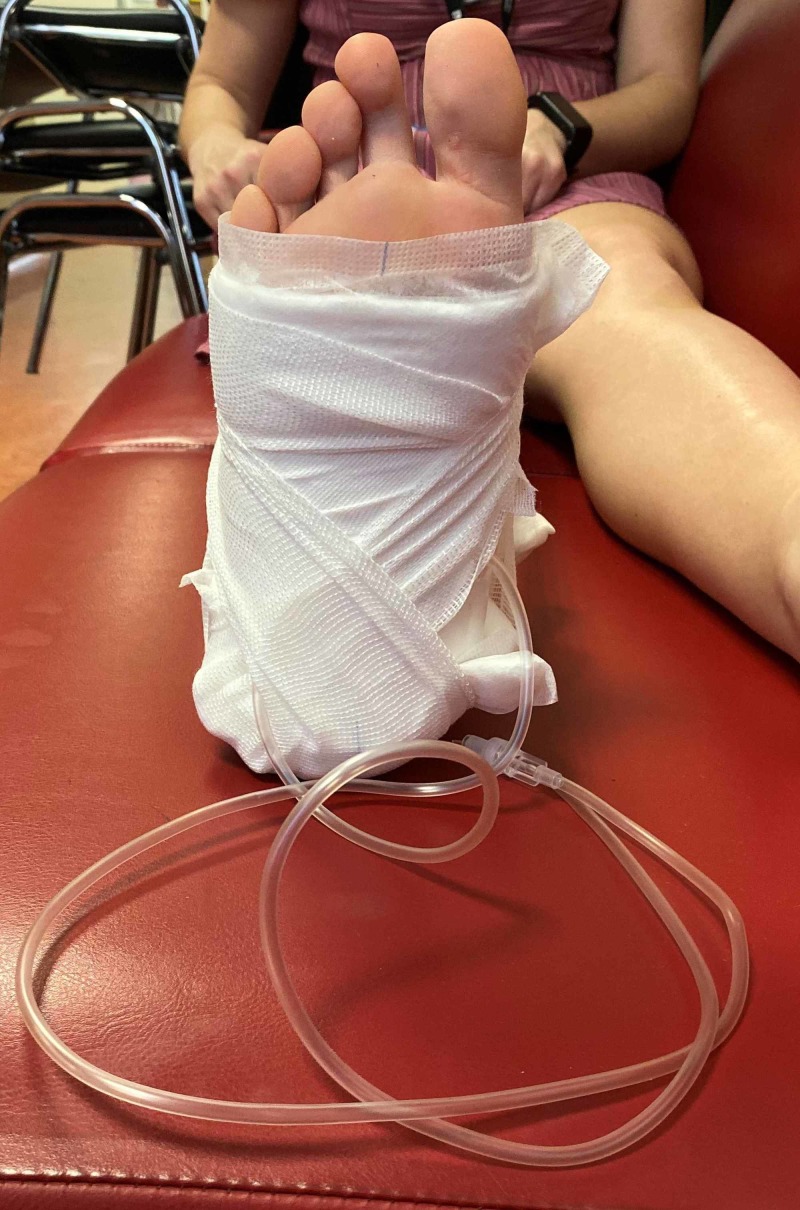
crepe bandage wrapped in a figure of eight around the bottom of the traction felt

The first crepe bandage is then wrapped around the foot and ankle in a figure-eight direction so that it covers the bottom of the padding and the ankle (Figure 5). This will hold the traction kit in place and also provide reinforcement to the padding while under the weight so the IV tubing is not dislodged when traction is applied.

**Figure 6:**
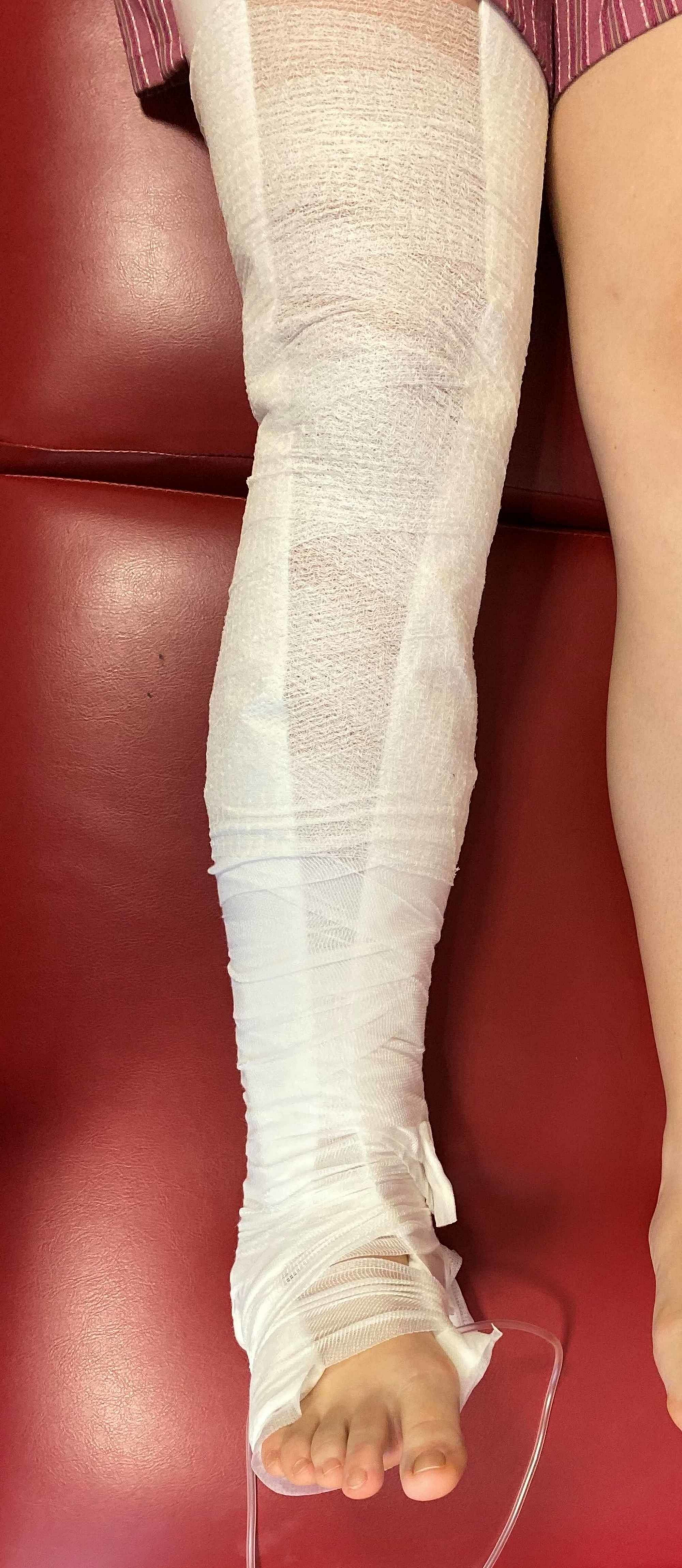
skin traction secured with crepe bandages

The second and third crepe are applied from ankle to above the knee in the same criss-cross method that is used for skin traction. Medical tape is used to secure the bandages into place (Figure 6). Once fully applied, bags of saline can be hung from the tubing over the end of the bed to provide traction.

**Figure 7:**
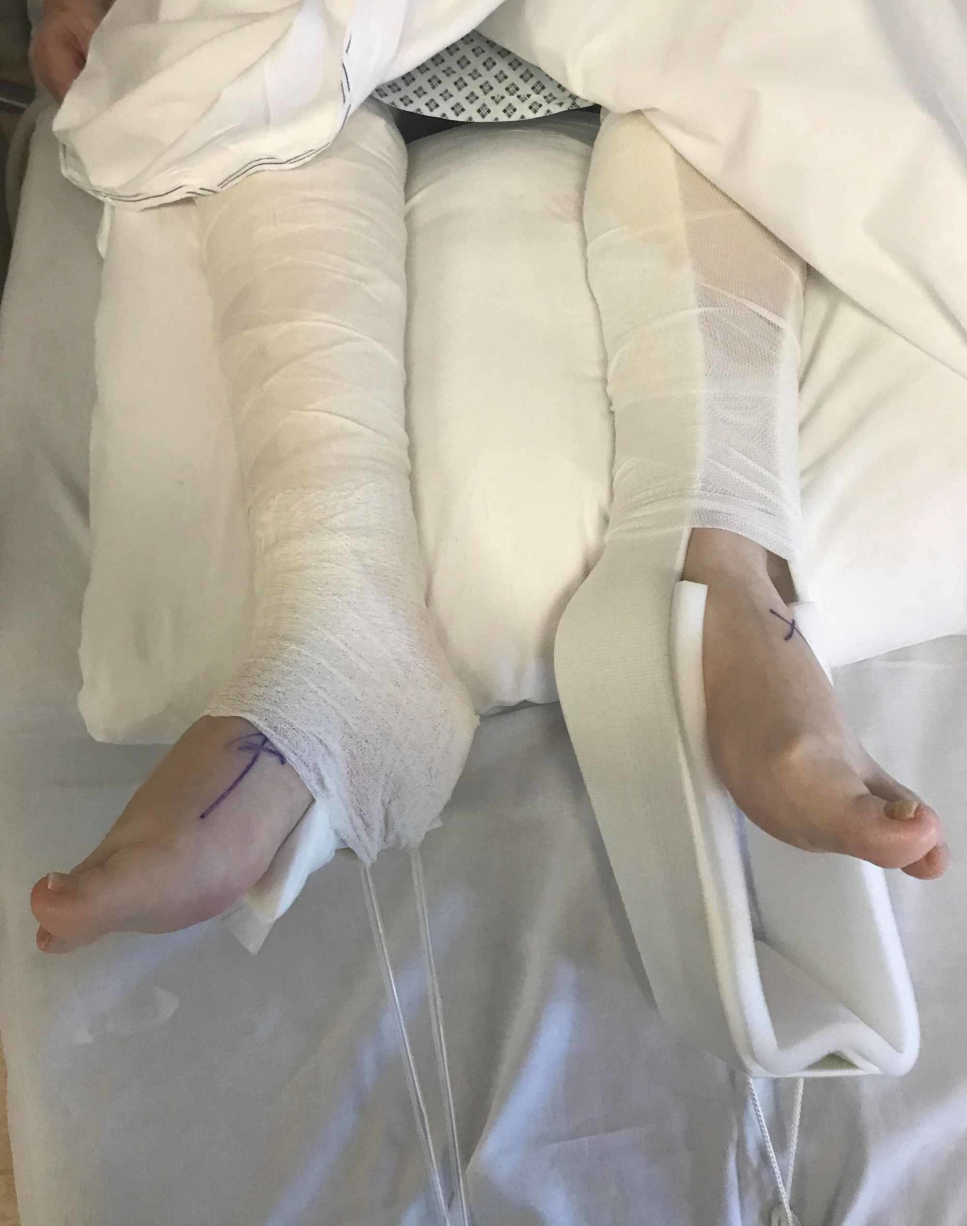
The completed skin traction kit Right leg: self-made kit using widely available hospital resources to achieve adequate traction while minimising the risk of pressure sores in a distal femur fracture Left leg: traditional ready-made skin traction kit that has slipped, offering inadequate skin traction

The IV fluid bags that are used as weights are then hung from the cut plastic IV tubing over the end of the bed. A finished example of adequate traction using this self-made kit is shown below (Figure 7). As with regular skin traction, the pulses should be checked after placement and the skin should be monitored regularly while the patient awaits definitive fixation.

## Discussion

Distal femur fractures are rare but significant injuries that routinely require definitive operative fixation [4]. They make up 0.4 percent of all fractures and 3% of femoral fractures [4]. As previously stated, two peaks of distal femur fractures exist; those in young people after high- energy, polytrauma incidents, usually associated with frontal collision mechanisms; and those in the elderly, which are more spiroid in nature, most likely due to low-energy trauma in osteoporotic bone [1-2]. Fixation is difficult in both epidemiological peaks due to the levels of communication associated with the younger cohort and the lack of quality bone stock in the elderly [2-4]. 

Although treatment has evolved during the past few years, distal femur fractures remain among the more challenging injuries for orthopaedic surgeons to manage [2-4]. This is due to their high complications rate, which is often associated with severe pain and functional disability. Distal femur fractures have a significant impact on the function of the knee, even when they are extra-articular. Any failure to restore the functional angles of the distal femur will result in long-term pathomechanics of the knee, directly impairing joint motion and stability [4]. Even after definitive fixation has been achieved, patients often face months of physiotherapy and rehabilitation in a bid to regain pre-morbid baseline function [1-2]. Previous studies examining distal femoral fixation in the elderly population have reported a 50% mortality at 5-year follow-ups and frequent loss of self-dependence, and only 18% of the patients have been found able to walk unaided [2].

Balanced skeletal traction is advised in patients for whom internal fixation will be definitive treatment, as it avoids excessive exertional forces of displaced fracture segments through the skin and soft tissue layers [3-4]. This is often achieved with Thomas Splints or ready-made skin traction kits such as Sterotrac (Steroplast Healthcare, Manchester, UK) or Tensoplast (BSN medical GmbH, Hamburg, Germany) kits pending definitive fixation. The availability of these kits is often sporadic and, in developing and resource-poor health systems, the kits may not be available at all. Furthermore, if a centre experiences high-volume levels of trauma, such as in a trauma centre or within a warzone, stock can be depleted rapidly.

Another issue associated with increased morbidity in the elderly is the heightened risk of pressure sores associated with this type of fracture. Elderly patients presenting with these types of fractures typically have a reduced physiological reserve [6]. Sarcopenia and frailty result in biological and physiological changes characterised by reduced muscle strength and functional dependence [6]. Distal femur fractures may result in long periods of immobilisation, which can lead to weight loss, loss of mobility and declined physiological reserve [6]. Sweating in the typical traction kit may produce moisture, which, when combined with prolonged immobilisation, will hasten the risk of developing a pressure sore. While padding is issued as part of the standard kit, this becomes ineffective if the traction slips as seen in Figure 7. Furthermore, the ready-made kits do not provide pressure relief for the heels, leaving them susceptible to damage.

The authors feel that this “do-it-yourself” skin traction technique provides equivalent traction compared to a traditional ready-made skin traction kit and can be used safely and effectively in the interim before definitive fixation to stabilise a distal femur fracture. It can be made by utilising widely and readily available common hospital resources. Furthermore, our technique provides increased protection to pressure areas for the patient due to the significant padding provided by the surgical pads, reducing the risk of pressure sores associated with traditional skin traction.

## Conclusions

This skin traction technique is made of common, inexpensive supplies and uses the same technique as regular skin traction application, making this method readily accessible to orthopaedic and emergency staff alike. This technique also provides patients with safe and comfortable traction while maintaining appropriate precautions against pressure sores while they await definitive fixation.
